# Ten-year radiographic and functional outcomes in rheumatoid arthritis patients in remission compared to patients in low disease activity

**DOI:** 10.1186/s13075-023-03176-7

**Published:** 2023-10-20

**Authors:** Adeline Ruyssen-Witrand, Gregory Guernec, Julia Dupont, Diane Lapuyade, Frédéric Lioté, Olivier Vittecoq, Yannick Degboé, Arnaud Constantin

**Affiliations:** 1 Rheumatology Centre, Toulouse University Hospital, Centre d’Investigation Clinique de Toulouse CIC1436, Inserm, Team PEPSS “Pharmacologie En Population cohorteS Et biobanqueS, Purpan Teaching Hospital, University of Toulouse 3, 1 Place du Dr Baylac, 31059 Toulouse, Cedex 9 France; 2 Inserm, Centre d’Epidémiologie Et de Recherche en Santé Des Populations, UMR1295, Inserm, Toulouse, France; 3 Rheumatology Centre, Toulouse University Hospital, Toulouse, France; 4 Université Paris Cité and Inserm UMR1132 Bioscar Hôpital Lariboisière and Service de Rhumatologie, Hôpital Saint-Joseph, Paris, France; 5 Department of Rheumatology and CIC-CRB1404, Normandie Univ, UNIROUEN, Rouen University Hospital, 76000 Rouen, France; 6 Rheumatology Center, Toulouse University Hospital, INFINITY, Toulouse Institute for Infectious and Inflammatory Diseases, INSERM U1291, CNRS U5051, University Toulouse 3, Toulouse, France

**Keywords:** Rheumatoid arthritis, Prognosis, Long-term outcomes, Remission, Disease activity

## Abstract

**Background:**

To compare the 10-year structural and functional prognosis between patients in sustained remission versus patients in sustained low disease activity (LDA) in early rheumatoid arthritis (RA).

**Methods:**

We included 256 patients from the ESPOIR cohort who fulfilled the 2010 ACR/EULAR criteria for RA and who were in sustained remission using the Simple Disease Activity Index (SDAI) score (*n* = 48), in sustained LDA (*n* = 139) or in sustained moderate to high disease activity (MDA or HDA, *n* = 69) over 10 years. The mTSSs progression over 10 years and the 10-year HAQ-DI scores were compared between the 3 groups. A longitudinal latent process mixed model was used to assess the independent effect of SDAI status over time on 10-year mTSS progression and HAQ-DI at 10 years.

**Results:**

Patients in sustained remission group were younger, had lower baseline HAQ-DI and mTSS scores and were less exposed to glucocorticoids, methotrexate or biologic disease-modifying anti-rheumatic drugs over 10 years. Patients in sustained remission had lower 10-year structural progression (variation of mTSS in the remission group: 4.06 (± 4.75) versus 14.59 (± 19.76) in the LDA group and 21.04 (± 24.08), *p* < 0.001 in the MDA or HDA groups) and lower 10-year HAQ-DI scores (10-year HAQ-DI in the remission group: 0.14 (± 0.33) versus 0.53 (± 0.49) in the LDA group and 1.20 (± 0.62) in the MDA or HDA groups, *p* < 0.001). The incidence of serious adverse events over 10 years was low, about 3.34/100 patient years, without any difference between the three groups.

**Conclusion:**

RA patients in sustained SDAI remission have better long-term structural and functional outcomes in comparison to patients in sustained LDA.

**Supplementary Information:**

The online version contains supplementary material available at 10.1186/s13075-023-03176-7.

## Background

The prognosis for rheumatoid arthritis (RA) has improved significantly over the past 20 years since the introduction of biologic disease-modifying anti-rheumatic drugs (bDMARDs) and the treat-to-target method associated with tight disease control strategies [1]. According to the latest EULAR (European League Against Rheumatisms) recommendations [1], the goal when introducing a DMARD in RA is to achieve remission at 6 months or at least to achieve a low disease activity. The EULAR and ACR (American College of Rheumatology) also recommend the use of the Simple Disease Activity Index (SDAI) tool or Boolean remission criteria to assess remission [2]. It is currently well demonstrated that achieving disease remission is associated with a better long-term structural and functional prognosis compared to sustained moderate or high activity [3–6]. However, there is little evidence in the literature of the impact of sustained remission on long-term prognosis in comparison to sustained low disease activity (LDA). Achieving sustained remission may lead to escalation therapies that may be responsible for side effects. In a previous study, we had shown that achieving SDAI remission 1 year after the introduction of a DMARD was associated with a better 3-year structural prognosis compared to achieving an LDA state in RA [7].

The main objective of this study was to compare the 10-year structural and functional prognosis between patients with sustained remission versus patients in sustained LDA in early RA. The secondary objective was to compare the incidence of serious adverse events at ten years between patients in sustained remission in comparison to patients in sustained LDA.

## Methods

### Patients

The analysis was based on patient data from the ESPOIR (Étude et Suivi des *Polyarthrites* Indifférenciées Récentes) cohort (NCT03666091). The characteristics of patients in the whole cohort have been previously reported [8]. Briefly, patients were included in the cohort between 2002 and 2005 if they had at least two occurrences of synovitis of small joints lasting for more than 6 weeks and less than 6 months, if they had not previously received any DMARD, and were suspected of having RA according to a rheumatologist. These patients were then followed prospectively every 6 months for the first 2 years and every year up to 10 years.

Demographic, clinical, biological characteristics and X-rays were performed at each visit.

Patients meeting the ACR/EULAR criteria during cohort follow-up were selected in this study (*N* = 646). Of these, only patients who made at least 60% of the in-between 10-year follow-up visits were selected.

The institutional review board (ethics committee of Montpellier, France, no. 020307) approved the protocol, and all patients provided written informed consent. This study was performed without direct patient and public involvement.

### Definition of the groups based on disease activity scores

Disease activity scores were expressed with SDAI and calculated as follows: TJC28 + SJC28 + patient global VAS (0–10 cm) + physician global VAS (0–10 cm) + CRP (mg/dL). We also used the DAS28-ESR criterion to repeat the analysis, calculated as follows: 0.56√(TJC28) + 0.28√(SJC28) + 0.70ln (ESR) + 0.014 (patient global VAS (0–100 mm)) [9].

Three different methods were used to carry out the groups based on disease activity status. We first sought to identify patients with an SDAI < 3.3 at all the visits comprised between the 1-year and the 10-year visits for the sustained remission group and patients with an SDAI comprised between 3.3 and 11 at all the visits comprised between the 1-year and 10-year visits. This method identified only seven patients in the sustained SDAI remission group and only one patient in the sustained SDAI LDA group and appeared as a limiting factor given the longitudinal studies considered. Since disease activity fluctuations are too frequently observed in the ESPOIR cohort, we have sought to build up these groups by modifying the usual thresholds of SDAI using two other approaches.

We first sought to classify patients according to their SDAI trajectory over the 10-year follow-up period. To do this, we have created several latent class models [10, 11] in order to group patients according to several SDAI trajectories over 10 years. Details on latent class models for identifying patient SDAI trajectories and results are described in the Supplementary material (see Supplementary Material Data S1 and Figure S1). After reviewing the results of the classification based on SDAI trajectories, we ultimately did not choose this method of classification because of the presence of too many misclassified patients (see Supplementary Material Table S1 for examples of misclassifications).

Using a second approach, we grouped patients according to their SDAI with predefined thresholds. The objective of defining these new SDAI thresholds was to allow for moderate disease activity fluctuations, with the final purpose of aggregating patients who mainly remained in sustained SDAI remission or in sustained SDAI LDA over the 10-year follow-up period. The values of these new SDAI thresholds were chosen from the tertiles of the SDAI scores obtained after a re-sampling method [12] (see Supplementary Material Table S2 and Supplementary Figure S2 for disease activity trajectories according to the groups). Patients with too-significant disease activity fluctuation over time were excluded. A sensitivity analysis was performed by modifying these disease activity thresholds. Details on the definition of SDAI thresholds and predefined choices to classify patients, as well as sensitivity analyses, are described in the Supplementary material (Supplementary Data S2, Supplementary Table S3).

The latter method defined three groups of patients: the group of patients in sustained SDAI remission (*N* = 48), a group of patients in sustained SDAI LDA (*N* = 139) and a group of patients with sustained SDAI moderate to high disease activity (*N* = 69). Thus, 271 patients were excluded from the analysis because they had too-large activity fluctuation to be classified among these three groups.

These group-building methods were repeated using the DAS28-ESR criterion (see Supplementary Tables S2 and S4 and Figure S3).

### Outcomes of interest

The two main outcomes were the 10-year structural progression defined on the change in the Total Sharp Score modified by Van der Heijde (mTSS) between the inclusion visit and the 10-year visit and the 10-year functional impairment expressed by the 10-year HAQ-DI (Health Assessment Questionnaire Disability Index). The details of key interpretation to assess mTSS have already been reported elsewhere [13].

The secondary outcome was the incidence of serious adverse events at 10 years, including severe infectious events defined by hospitalisation or parenteral antibiotic therapy at 10 years, major cardiovascular events (MACEs), including myocardial infarction, stroke, and cardiovascular mortality, thromboembolism events (including pulmonary embolisms and deep venous thrombosis), neoplasia and overall mortality.

In addition to the disease activity criteria collected at each visit (SDAI, DAS28, VS, CRP, patient global disease activity assessed by a visual analogic scale (PatGlob), number of swollen and tender joints), demographic data (sex, age at diagnosis, smoking), disease characteristics (duration of evolution of symptoms at inclusion, presence of rheumatoid factors or ACPA at inclusion, presence of erosion at inclusion), as well as treatments used during follow-up, including synthetic DMARDs and biological DMARDs, and the use of corticosteroids. In case of missing data on these covariables, a standard multiple imputation approach was performed [14].

### Statistical analysis

The characteristics of the patients included in this analysis were described according to the disease activity group. Qualitative variables were expressed in numbers and percentages, and quantitative variables were expressed in averages and standard deviations. The incidence of serious adverse events at 10 years was expressed as the number of events per 100 patient years.

The comparison of the variation of the 10-year mTSS scores and the 10-year HAQ-DI scores according to the groups based on SDAI or DAS28-ESR was carried out in variance analysis by ANOVA. The proportion of serious adverse events at 10 years was compared using a univariate *χ*^2^ test.

A longitudinal latent process mixed model [15] with repeated data in latent classes, including repeated activity measures over time, was performed to predict the structural progression expressed by the variation of the mTSS score and included the following covariates: disease activity group based on SDAI or DAS28-ESR, age, sex, smoking, duration of symptoms, centre of inclusion, presence of rheumatoid factors and/or ACPA, presence of erosion at diagnosis and the exposure to csDMARDs, bDMARDs or corticosteroids over the 10-year follow-up period. The same model was repeated, using the variables to explain the HAQ-DI score at 10 years. Slope differences over the entire duration of follow-up (10 years), and mean differences after 10 years between groups of DAS28-ESR were estimated by testing appropriate linear combinations from the final model parameters [16]. The Wald tests related to these contrasts were implemented in the R software via the WaldMult function of the lcmm package.

Finally, the association between disease activity and the risk of serious adverse events at ten years was evaluated in a Cox model [17, 18]. The covariables used in the model were disease activity group based on SDAI or DAS28-ESR, sex, age, smoking, use of corticosteroids during follow-up (corticosteroids, synthetic or biological background DMARD), comorbidities, rheumatoid factors and/or ACPA, the presence of erosions at diagnosis, the HAQ-DI score and the CRP over time.

Details on the constitution of the multivariate model for the structural, functional and safety outcomes are outlined in the Supplementary material (Supplementary Data S3).

## Results

### Patients

**Fig. 1:**
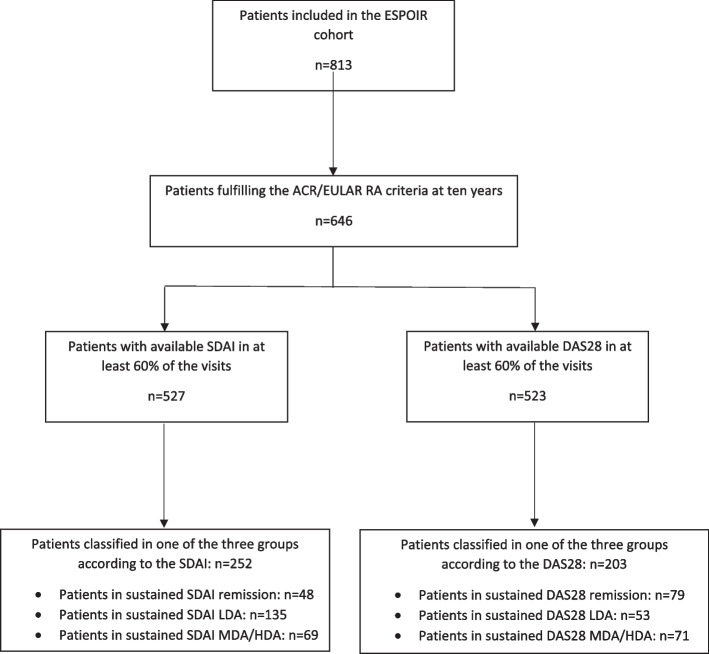
Flow chart of patients included in this study. ACR/EULAR, American College of Rheumatology/European; RA, rheumatoid arthritis; SDAI, Simple Disease Activity Index; DAS28, Disease Activity Score with 28 joints; LDA, low disease activity; MDA, moderate disease activity; HDA, high disease activity

The flow chart of selected patients is presented in Fig. 1.

The characteristics of the patients at inclusion, as well as the treatments taken over time, are summarised in Table 1 for the groups defined by the SDAI and Supplementary Table S5 for the groups defined by the DAS28.

**Table 1:** Patient inclusion characteristics and treatments taken over time by their SDAI group during the 10-year follow-up in the ESPOIR cohort

Patient characteristics	REM *N* = 48	LDA *N* = 135	MDA or HDA *N* = 69	*p*-value
**Baseline characteristics**				
Age, years, mean (SD)	45.0 (12.1)	52.2 (10.9)	49.7 (11.6)	< 0.01
Number of female subjects (%)	38 (79)	101 (75)	57 (83)	NS
Disease duration, months, mean (SD)	7.8 (10.3)	7.5 (9.2)	8.5 (9.7)	NS
RF, number (%)	24 (50)	58 (43)	39 (57)	NS
ACPA, number (%)	19 (40)	69 (51)	26 (38)	NS
Typical erosions, number of patients (%)	20 (42)	73 (54)	40 (58)	NS
ESR, mean (SD)	30.2 (27.5)	30.7 (25.8)	32.1 (25.8)	NS
CRP, mean (SD)	23.8 (33.2)	23.1 (32.3)	29.0 (44.8)	NS
mTSS, mean (SD)	1.49 (1.95)	3.38 (5.25)	5.08 (8.03)	< 0.05
HAQ-DI, mean (SD)	0.78 (0.59)	0.94 (0.65)	1.23 (0.68)	< 0.001
Smokers, number (%)	29 (60)	55 (40)	34 (49)	NS
**Ten-year characteristics**				
Corticosteroids, number of patients (%)	33 (69)	112 (83)	60 (87)	< 0.05
Corticosteroid cumulative dose, gr, mean (SD)	1.3 (3.5)	4.5 (6.9)	7.4 (11.1)	< 0.001
DMARDs, number of patients (%)	36 (75)	120 (89)	64 (95)	0.01
DMARDs exposure duration, months, mean (SD)	45.6 (115.4)	89.6 (42.9)	77.6 (43.2)	< 0.001
Methotrexate, number of patients (%)	29 (60)	111 (82)	58 (84)	< 0.01
Methotrexate exposure duration, months, mean (SD)	40 (46.5)	75 (49.6)	60.7 (48.7)	< 0.001
bDMARDs, number of patients (%)	6 (13)	32 (24)	36 (92)	< 0.001
bDMARDs exposure duration, months, mean (SD)	7.5 (24.3)	13.4 (28.3)	27.7 (35.9)	< 0.001
10-year mTSS, mean (SD)	4.06 (4.75)	14.59 (19.76)	21.04 (24.08)	< 0.001
10-year HAQ, mean (SD)	0.14 (0.33)	0.53 (0.49)	1.20 (0.62)	< 0.001

*REM* patients in sustained remission, *LDA* patients in sustained low disease activity, *MDA* patients in moderate disease activity, *HDA* patients in sustained high disease activity, *SD* Standard deviation, *RF* rheumatoid factor, *ACPA* anti-citrullinated peptides antibodies, *mTSS* van der Heijde modified Total Sharp Score, *HAQ-DI* Health Assessment Questionnaire Disability Index, *DMARDs* disease-modifying anti-rheumatic drugs, *bDMARDs* biologic disease-modifying anti-rheumatic drugs

Patients in sustained remission were younger (45 years for patients in the remission group compared to 52 years for patients in the LDA group and 49 years for patients in the MDA or HDA groups, *p* < 0.01) at inclusion in the ESPOIR cohort and had a lower baseline HAQ-DI (0.78 for patients in the remission group versus 0.94 for patients in the LDA group and 1.23 for patients in the MDA and HDA groups, *p* < 0.001) and mTSS score (1.49 for patients in remission compared to 3.38 for patients in LDA and 5.08 for patients in the MDA and HDA groups, *p* < 0.05). They were also less exposed to corticosteroids (69% of patients in remission compared to 83% for patients in LDA and 87% for patients in the MDA or HDA groups, *p* < 0.05) or DMARDs (75% of patients in the remission group compared to 89% in patients in LDA and 95% in patients in the MDA or HDA groups, *p* < 0.01) during the 10 years of follow-up. Unsurprisingly, patients in sustained MDA or HDA were more exposed to bDMARD treatments (92% of patients in MDA or HDA compared to 13% of patients in the remission group and 24% of patients in the LDA group, *p* < 0.001). Details on DAS28 analyses are provided in the Supplementary material (Supplementary Data S4 and Table S5).

Univariate analyses revealed that patients in sustained remission had lower 10-year mTSS scores and 10-year HAQ-DI scores compared to patients in sustained LDA and patients in sustained MDA or HDA (10-year mTSS mean in the remission group: 4.06 (SD: 4.75), compared to 14.59 (19.76) in the LDA group and 21.04 (24.08) in the MDA or HDA group, *p* < 0.001, 10-year HAQ-DI in the remission group: 0.14 (SD: 0.33) compared to 0.53 (SD: 0.49) in the LDA group and 1.20 (SD: 0.62), *p* < 0.001).

### Association between disease activity groups and variation of mTSS within ten years (Table 2)

**Table 2:** Multivariate analysis assessing the 10-year mTSS progression and 10-year HAQ-DI: final models using SDAI as disease activity score

Outcome	Variables	Wald test	*p*-value
Ten-year mTSS progression	Centre	16.5	NS
	Baseline erosions	59.9	< 0.0001
	SDAI group	8.1	0.01
	SDAI group* time	4.5	NS
	SDAI*intercept	0.6	NS
	ACPA	0.4	NS
	ACPA*time	30.6	< 0.0001
	ACPA*intercept	7.3	0.006
	Time	1.5	NS
	Intercept	1.1	NS
Results of the contrast method	Final model:		
	LDA versus remission	3.2	< 0.01
	MDA or HDA versus remission	4.5	< 0.0001
	MDA or HDA versus LDA	2.1	< 0.05
Ten-year HAQ-DI	RF	8.3	< 0.01
	bDMARD	11.9	< 0.001
	SDAI group	57.5	< 0.0001
	SDAI group*time	98.9	< 0.0001
	SDAI group*intercept	72.1	< 0.0001
	DMARDs use	0.04	NS
	DMARDs use*time	6.1	< 0.05
	DMARDs use*intercept	7.5	< 0.01
	Methotrexate use	0.1	NS
	Methotrexate*time	1.7	NS
	Corticosteroids use	3.9	< 0.05
	Corticosteroids use*time	6.7	< < 0.01
	Corticosteroids use*intercept	5.8	< 0.05
	Time	12.7	< 0.001
	Intercept	9.5	< 0.01
Results of the contrast method	Final model:		
	LDA versus remission	5.2	< 0.0001
	MDA or HDA versus remission	9.0	< 0.0001
	MDA or HDA versus LDA	5.6	< 0.0001

*mTSS* van der Heijde modified Total Sharp Score, *SDAI* Simple Disease Activity Index, *ACPA* anti-citrullinated peptide antibodies, *DMARD* disease-modifying anti-rheumatic drugs, *bDMARDs* biologic disease-modifying anti-rheumatic drugs. The included variables tested with univariate then multivariate analyses included: disease activity group based on SDAI, age, sex, smoking, duration of symptoms, centre of inclusion, presence of rheumatoid and/or ACPA factors, presence of erosion at diagnosis, synthetic background treatment, corticosteroids and bDMARD use

**Fig. 2:**
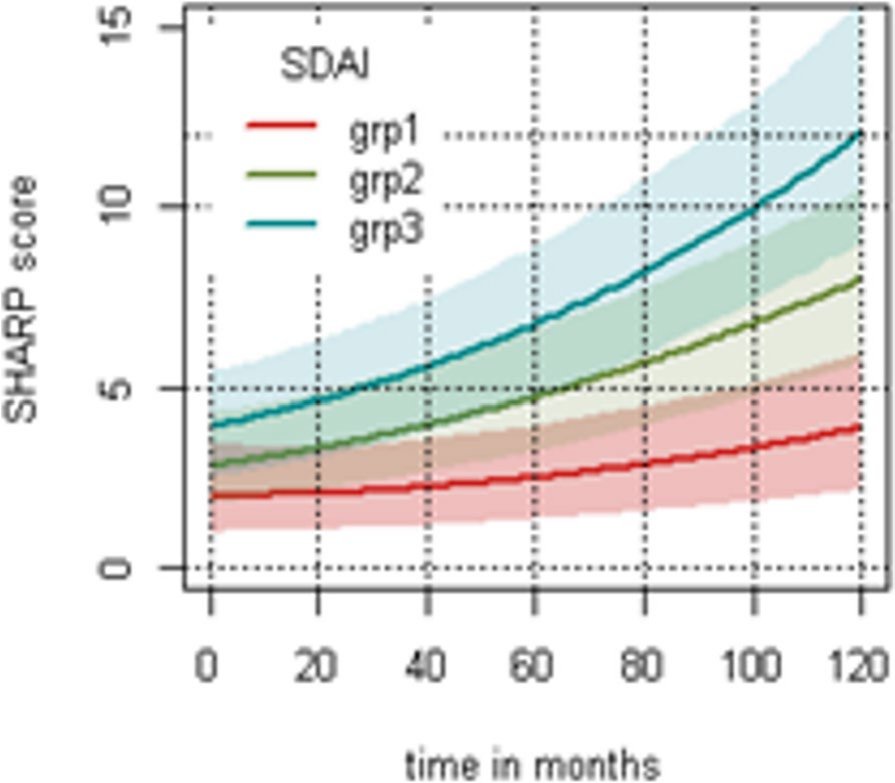
Predicted mTSS progression over time according to the SDAI group. Group 1: sustained SDAI remission group. Group 2: sustained SDAI LDA group. Group 3: sustained MDA or HDA group

After multivariate analysis, using contrast method, we clearly identified a difference in mTSS progression between patients in sustained SDAI remission and sustained SDAI LDA (*p* = 0.001), as well as between patients in sustained SDAI remission and in sustained SDAI MDA or HDA (*p* < 0.0001) (Fig. 2). Patients in sustained SDAI LDA also had significantly less structural progression than patients in MDA or HDA, even if the association was lower (*p* = 0.03). The other variables associated with structural progression were erosions at baseline (*p* < 0.0001) and ACPA with interaction with time (*p* < 0.0001) (Table 2).

All these analyses were repeated using DAS28-ESR as a disease activity tool and are available in the Supplementary material (Supplementary Figure S6, Supplementary Table S7). After multivariate analyses, we identified a significant difference of 10-year mTSS progression in patients in sustained DAS28 remission compared to sustained DAS28-ESR MDA or HDA (*p* < 0.0001) and in patients in sustained DAS28-ESR LDA versus patients in sustained DAS28-ESR MDA or HDA (*p* < 0.001), but we could not find a difference between patients in sustained DAS28-ESR remission versus in sustained DAS28-ESR LDA (see Supplementary Figure S4).

### Association between disease activity groups and 10-year HAQ-DI (Table 2)

**Fig. 3:**
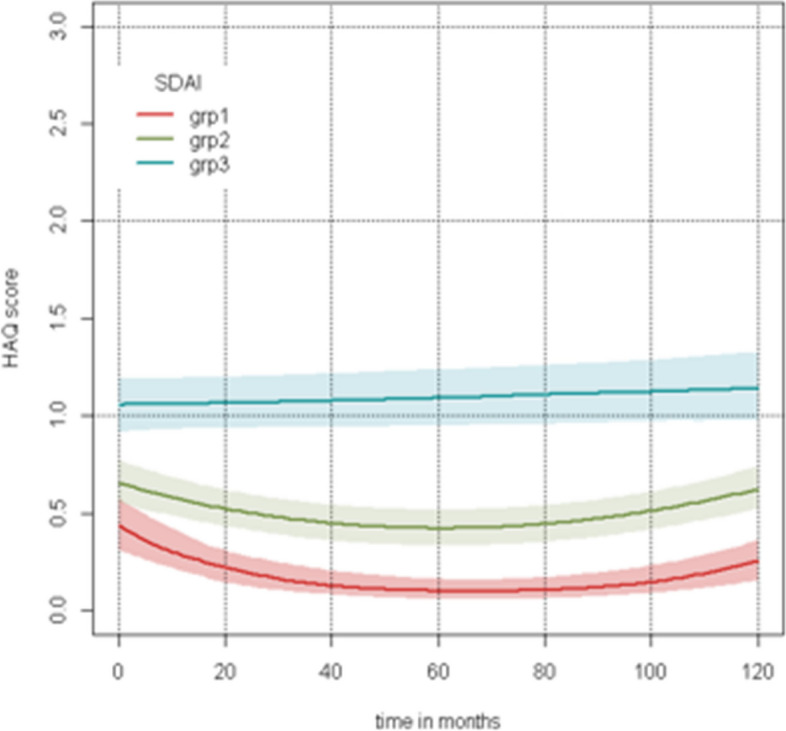
Predicted 10-year HAQ-DI according to the SDAI group. Group 1: sustained SDAI remission group. Group 2: sustained SDAI LDA group. Group 3: sustained MDA or HDA group

After multivariate analysis, the model identified a difference of 10-year HAQ-DI between patients in sustained SDAI remission and sustained SDAI LDA (*p* < 0.0001), as well as between patients in sustained SDAI remission and in sustained SDAI MDA or HDA (*p* < 0.0001) and between patients in sustained LDA versus sustained MDA or HDA (*p* < 0.0001) (Fig. 3). The other variables associated with structural progression were rheumatoid factors (*p* < 0.01), bDMARD exposure (*p* < 0.0001), any DMARD exposure over time (*p* < 0.05) and corticosteroid exposure (*p* < 0.001) (Table 2).

All these analyses were repeated using DAS28-ESR as a disease activity tool and are available in the Supplementary material (Supplementary Figure S8, Supplementary Table S7). The model identified a significant difference among 10-year HAQ in patients in sustained DAS28-ESR remission compared to sustained DAS28-ESR LDA (*p* < 0.001), in patients in sustained DAS28-ESR remission compared to sustained DAS28-ESR MDA or HDA (*p* < 0.0001) and in patients in sustained DAS28-ESR LDA versus patients in sustained DAS28 MDA or HDA (*p* < 0.001) (Supplementary Figure S8, Table S9).

### Sensitivity analyses

To verify the consistency of the results, we performed a sensitivity analysis, changing the threshold definition of the three SDAI groups and DAS28 groups. The description of the sensitivity analysis is provided in the Supplementary material (Supplementary Data S4, Supplementary Tables S9, S10, S11 and S12). Overall, after changing the definition of the groups, the results remained consistent with the primary analysis.

### Safety

We analysed the 10-year serious adverse events according to the three groups (Table 3). In the whole population, the incidence of serious adverse events was about 3.34/100 patient years, including serious infections with an incidence of 1.23/100 patient years and MACEs with an incidence of 0.44/100 patient years. We did not identify any differences between groups (Table 3 for groups based on SDAI, Supplementary Table S13 for groups based on DAS28-ESR).

**Table 3:** Ten-year serious adverse events according to the three groups of SDAI: 10-year cumulative incidence for 100 patient years

	All population (2032 PY)	REM (361 PY)	LDA (1128 PY)	MDA or HDA (532 PY)
Serious adverse events	3.34	3.60	3.01	4.14
Serious infections	1.23	1.93	0.97	1.31
Neoplasia	1.23	1.10	1.24	1.32
MACEs	0.44	0.55	0.18	0.94
Thromboembolism events	0.44	0.83	0.27	0.56
Death	0.49	0	0.53	0.75

*REM* patients in sustained remission, *LDA* patients in sustained low disease activity, *MDA* patients in moderate disease activity, *HDA* patients in sustained high disease activity, *PY* patient years, *MACEs* major cardiovascular events; all comparisons were non-significant

Due to the low number of events over 10 years, we only considered the overall 10-year serious adverse events for the multivariate analysis (see Supplementary Material Table S14). Disease activity according to the three groups was not associated with 10-year serious adverse events in the Cox model (see details in Supplementary Material Table S14).

## Discussion

The aim of this study was to compare 10-year structural progression and 10-year functional impairment between a group of patients in sustained remission and a group with patients in sustained LDA in an early RA population. The study showed a significant difference in 10-year structural progression and 10-year HAQ-DI between patients in SDAI sustained remission in comparison to patients in SDAI sustained LDA. When DAS28-ESR was the disease activity tool used to classify the patients, we did not identify a significant difference in 10-year structural progression between patients in sustained remission compared to patients in sustained LDA, while a difference was observed in 10-year HAQ-DI between these two groups. Ten-year serious adverse events were rare in this population, and we could not observe any difference between the two groups.

To our knowledge, this is the first study assessing the impact of sustained remission in comparison with sustained LDA on long-term outcomes in a real-life setting. We previously showed in the same cohort that patients in SDAI remission at 1 year had better structural outcomes at 3 years than patients in SDAI LDA. However, in our study, we could not show better HAQ-DI scores at 3 years in patients in SDAI remission at 1 year in comparison with patients in SDAI LDA. Furthermore, we were unable to show any differences between the two groups when DAS28-ESR was used as a disease activity score to classify patients [7]. Nikiphorou et al. [19] aimed to compare 5-year structural, functional and quality of life outcomes between a group of patients with mean DAS28 over the first 5 years that was below 2.6 (mean remission group) with a group of patients with mean DAS28 over the first five years that was between 2.6 and 3.2 (mean LDA group) in two cohorts of early RA (ERAN and ERAS cohorts). In this study, the patients were classified by mean DAS28 scores over 5 years without taking into account disease activity fluctuations over time. Indeed, 51% of patients in DAS28 remission were consistently classified in the remission group over the five first years, while only 12% of the patients in DAS28 LDA were consistently classified in the LDA group. Nevertheless, it could be seen with this classification that patients in the remission group had better functional and structural outcomes at 5 years than patients classified in the low disease activity group. It also compared patients in sustained Boolean remission at 1 and 2 years to patients in sustained DAS28 remission or LDA at 1 and 2 years and could show better functional, quality of life and structural outcomes at 5 years.

In our study, we selected patients with homogeneous disease activity trajectories to build the sustained remission or LDA groups, taking into account the possibility of mild disease activity fluctuations over time as is frequently observed in observational studies [20]. We decided to include patients with few values reasonably outside the typical thresholds of remission or LDA in a limited number of visits. With our method, we reduced the level of disease activity fluctuation and misclassification. However, we had to deal with a small number of patients with stable disease activity, who had at least 60% of available data over the 10 years and thus excluded around 2/3 of the patients of the ESPOIR cohort. This stringent selection process could lead to rare patient profiles with stable disease activity over time that may sound artificial and unrealistic. However, by including stable disease patients, we wanted to demonstrate long-term differences that could be explained by the disease activity status and not by fluctuation of disease activity. We had to make a compromise between a rigid definition of sustained remission or LDA and a sufficient number of patients to perform statistical comparisons. As the pre-defined rules were subjective, we also performed sensitivity analyses changing the pre-defined rules. We could see that when we repeated the analysis with new rules of classification, the results remained the same, showing the consistency of our findings. However, the rules for classifying the patients applied to the dataset of ESPOIR cohort and are not supposed to be extrapolated to other populations.

In our study, we identified a difference in 10-year radiographic progression between patients in SDAI sustained remission in comparison with patients in sustained SDAI LDA but not between patients in DAS28-ESR sustained remission in comparison with patients in sustained DAS28-ESR LDA (see Supplementary material). These findings are consistent with our previous analysis on the 3-year outcomes where no difference was observed when using the DAS28-ESR tool [7]. We also showed a difference in 10-year functional outcomes. This finding is consistent with expert guidelines that recommend using the ACR/EULAR definition of remission, including SDAI remission or Boolean remission criteria, the most stringent remission criteria instead of other remission tools and a better disease activity target as it is associated with less long-term radiographic changes and better functional outcomes [21, 22]. Indeed, residual inflammation is often observed in patients in DAS28-ESR remission that could explain long-term radiographic changes in some patients [23, 24].

The impact of achieving remission in comparison with moderate or high disease activity on long-term outcomes has been already demonstrated in many studies [3–5, 25, 26]. Here, we confirmed that patients in sustained SDAI or DAS28-ESR remission had less structural progression over 10 years and less functional impairment at 10 years.

We did not identify any differences of 10-year serious adverse events between the three groups, but this result needs to be interpreted with caution, because we observed very few 10-year serious adverse events in our population. As associations between disease activity and cardiovascular risk [27, 28], serious infection risk [29, 30], malignancy risk [31] and mortality [27, 28] were previously shown, we could expect less serious adverse events in the group of patients in sustained remission. However, our limited number of events prevent us from demonstrating this.

Our study has several limitations. Firstly, we excluded a large number of patients with missing data or disease activity fluctuation and the results can thus be extrapolated only to patients with stable disease activity. Secondly, as it is an observational study, we did not demonstrate here a causal effect between the disease activity status and long-term outcomes. Indeed, many factors could explain such a difference, including treatment strategies, centre effect, disease characteristics or patient characteristics. We tried to identify confusion factors through multivariable analyses, including patient and disease characteristics and treatment, but we cannot be sure if a remaining confusion factor has an impact on our results. Finally, in the ESPOIR cohort, patients had a follow-up visit every 6 months over the first 2 years, then yearly. Thus, we could only use the disease activity scores collected at these scheduled visits and patients may have had disease activity fluctuation between two visits that could not be captured. We assume with our method that it may not be the case that a patient in remission at two visits is also in remission during the whole year.

However, our study has several strengths. Firstly, the analyses are based on the ESPOIR cohort, which is one of the largest inception cohorts of patients with early RA where patients were prospectively followed, with scheduled visits where a lot of data could be systematically collected over ten years. Sixty-four percent of patients included in the ESPOIR cohort completed the 10-year visit, with no baseline differences between patients followed in the cohort and loss of follow-up patients [8]. RA patients were routinely managed according to the standard of care reflecting real life. Structural progression was assessed by a central interpretation performed by multi-reader assessment [13] and the database was rigorously built and monitored.

## Conclusion

This study identified a clear association between sustained remission and long-term structural and functional outcomes in comparison to patients in sustained low disease activity, without any differences in safety. This study gives additional data about the importance of targeting sustained SDAI remission when treating an RA patient.

### Supplementary Information

**Additional file 1:** **Supplementary Data S1.** Grouping patients according to disease activity state. **Supplementary Data S2.** Sensitivity analysis. **Supplementary Data S3.** Multivariate analysis. **Supplementary Data.** Sensitivity analyses. **Supplementary Data S4.** When using DAS28-ESR to assess disease activity, comparative analyses showed that patients classified in sustained remission according to DAS28-ESR were females in 70% of cases compared to 88% in the sustained LDA group and 92% in the MDA or HDA groups (*p* < 0.001) and had shorter disease duration (mean duration in remission group: 6.3 months (SD: 9.0) compared to 8.8 months (SD: 7.5) in the LDA group and 8.4 months (SD: 10.1) in the MDA or HDA groups, *p* < 0.001) and lower ESR (mean ESR in remission group: 24 (SD: 22) compared to 36 (SD: 28) in the LDA group and 39 (SD: 26) in the MDA or HDA groups, *p* < 0.001 while CRP levels were comparable across the three groups. Baseline HAQ scores were comparable between patients in sustained remission and LDA and lower than patients in the MDA or HDA groups (mean HAQ in the remission group: 0.86 (SD: 0.61) and 0.88 (SD: 0.64) in the LDA group compared to 1.28 (SD: 0.69) in the MDA or HDA groups, *p* < 0.001). Patients in the sustained remission group were less exposed to corticosteroids, DMARDs and bDMARDs during the follow-up in the cohort compared to patients in sustained LDA and sustained MDA or HDA (see Table S1). Univariate analyses revealed that patients in sustained remission had lower ten-year mTSS scores and ten-year HAQ scores compared to patients in sustained LDA and patients in sustained MDA or HDA (ten-year mTSS mean in remission group: 47.94 (10.75), compared to 12.18 (16.66) in the LDA group and 22.66 (25.19) in the MDA or HDA groups, *p* < 0.001, ten-year HAQ in remission group: 0.24 (0.38) compared to 0.58 (0.49) in the LDA group and 1.21 (0.68), *p* < 0.001). **Figure S1.** Profile with 95% confidence intervals of each group of patients provided by the optimal lcmm model. The right column provides the composition of each cluster in time by levels of SDAI score. (‘/number’ corresponds to the number of patients by cluster). **Supplementary Figure S2.** Individual patient trajectory profiles are represented in the graphs below, according to different groups. SDAI trajectories in the whole cohort and in the three groups (group 1= remission, group 2 = LDA, group 3 = MDA or HDA group). **Supplementary Figure S3.** DAS28 trajectories in the whole cohort and in the three groups (group 1 = remission, group 2 = LDA, group 3 = MDA or HDA group). **Supplementary Figure S6.** Profiles (with 95% confidence intervals) of mTSS trajectories over ten years using the DAS28-ESR score for disease activity-based groups. **Supplementary Figure S8.** Results of the modelisation of HAQ evolution over ten years using the DAS28-ESR score for disease activity-based groups. **Supplementary Table S1.** Example of SDAI. **Table S2.** Details of the bootstrapped tertiles including validity thresholds (standard method) by disease activity states for SDAI (a) and DAS28 (b). HDA: high disease activity, MDA: moderate disease. **Table S3.** SDAI group definition sensitivity analysis. **Table S4.** DAS28-ESR group definition sensitivity analysis. **Table S5.** Inclusion patient characteristics and treatments taken over time by their DAS28-ESR group during the ten-year follow-up in the ESPOIR cohort. **Table S7.** Multivariate analysis assessing the ten-year mTSS progression and ten-year HAQ using DAS28-ESR as disease activity score: Wald tests related to the parameter estimates and contrast of the final model. **Table S9.** Sensitivity analysis by modifying the rules of the definition of the three groups based on SDAI as disease activity score. Comparison of ten-year mTSS progression across the three groups using contrasts from the final model parameters. **Table S10.** Sensitivity analysis by modifying the rules of the definition of the three groups based on SDAI as disease activity score. Comparison of ten-year HAQ across the three groups using contrasts from the final model parameters. **Table S11.** Sensitivity analysis by modifying the rules of the definition of the three groups based on DAS28-ESR as the disease activity score. Comparison of ten-year mTSS progression across the three groups using contrasts from the final model parameters. **Table S12.** Sensitivity analysis by modifying the rules of the definition of the three groups based on DAS28-ESR as the disease activity score. Comparison of ten-year HAQ across the three groups using contrasts from the final model parameters. **Table S13.** Ten-year serious adverse events according to the three groups of DAS28-ESR: ten-year incidence for 100 patient years. **Table S14.** Hazard ratios of optimal Cox models to predict ten-year serious adverse events risk with SDAI (a) or DAS28-ESR (b) as rheumatoid arthritis activity scale (N(a) = 252; N(b) = 203).

## Data Availability

All of the individual participant deidentified data collected in the ESPOIR cohort, as well as the data dictionary can be available for further analyses after submitting a project to the scientific committee of the ESPOIR cohort (cohorte.espoir@gmail.com).

## Abbreviations

ACR/EULAR: American College of Rheumatology/European
DAS28: Disease Activity Score with 28 joints
ESPOIR: Étude et Suivi des Polyarthrites Indifférenciées Récentes
HAQ-DI: Health Assessment Questionnaire Disability Index
HDA: High disease activity
LDA: Low disease activity
MACE: Major cardiovascular events
MDA: Moderate disease activity
mTSS: Modified Total Sharp Score
RA: Rheumatoid arthritis
REM: Remission
SDAI: Simple Disease Activity Index

## References

[1] Smolen JS, Landewe RBM, Bijlsma JWJ (2020). EULAR recommendations for the management of rheumatoid arthritis with synthetic and biological disease-modifying antirheumatic drugs: 2019 update. Ann Rheum Dis.

[2] Felson DT, Smolen JS, Wells G (2011). American College of Rheumatology/European League Against Rheumatism provisional definition of remission in rheumatoid arthritis for clinical trials. Arthritis Rheum.

[3] Kavanaugh A, Fleischmann RM, Emery P (2013). Clinical, functional and radiographic consequences of achieving stable low disease activity and remission with adalimumab plus methotrexate or methotrexate alone in early rheumatoid arthritis: 26-week results from the randomised, controlled OPTIMA study. Ann Rheum Dis.

[4] Klarenbeek NB, Koevoets R, van der Heijde DM (2011). Association with joint damage and physical functioning of nine composite indices and the 2011 ACR/EULAR remission criteria in rheumatoid arthritis. Ann Rheum Dis.

[5] Schipper LG, van Hulst LT, Grol R, van Riel PL, Hulscher ME, Fransen J (2010). Meta-analysis of tight control strategies in rheumatoid arthritis: protocolized treatment has additional value with respect to the clinical outcome. Rheumatology (Oxford).

[6] van Tuyl LH, Felson DT, Wells G, Smolen J, Zhang B, Boers M (2010). Evidence for predictive validity of remission on long-term outcome in rheumatoid arthritis: a systematic review. Arthritis Care Res.

[7] Ruyssen-Witrand A, Guernec G, Nigon D (2015). Aiming for SDAI remission versus low disease activity at 1 year after inclusion in ESPOIR cohort is associated with better 3-year structural outcomes. Ann Rheum Dis.

[8] Combe B, Rincheval N, Berenbaum F (2021). Current favourable 10-year outcome of patients with early rheumatoid arthritis: data from the ESPOIR cohort. Rheumatology (Oxford).

[9] Aletaha D, Landewe R, Karonitsch T (2008). Reporting disease activity in clinical trials of patients with rheumatoid arthritis: EULAR/ACR collaborative recommendations. Ann Rheum Dis.

[10] McCulloch CE, Lin H, Slate EH, Turnbull BW (2002). Discovering subpopulation structure with latent class mixed models. Stat Med.

[11] McCutcheon AL. Latent class analysis. Sage; 1987.

[12] Efron B, Tibshirani R. An introduction to the bootstrap. Boca Raton: Chapman & Hall/CRC Monographs on Statistics and Applied Probability; 1993.

[13] Gandjbakhch F, Granger B, Freund R (2017). Multireader assessment as an alternative to reference assessment to improve the detection of radiographic progression in a large longitudinal cohort of rheumatoid arthritis (ESPOIR). RMD Open.

[14] van Buuren S. Multiple imputation of discrete and continuous data by fully conditional specification. Stat Methods Med Res. 2007;16(3):219–42. 10.1177/0962280206074463. 10.1177/0962280206074463 17621469

[15] Proust-Lima C, Amieva H, Jacqmin-Gadda H. Analysis of multivariate mixed longitudinal data: a flexible latent process approach. Br J Math Stat Psychol. 2012;66(3):470–87. 10.1111/bmsp.12000 23082854

[16] Agresti A. Categorical data analysis. New York: Wiley; 1990.

[17] Cox DR. Regression models and life-tables (with discussion). J R Stat Soc B. 1972;34:187–220.

[18] Kalbfleisch J, Prentice R. The statistical analysis of failure time data, second edition. Book Series: Wiley Series in Probability and Statistics. Wiley; 2002. ISBN:9780471363576.

[19] Nikiphorou E, Norton SJ, Carpenter L (2020). Remission vs low disease activity: function, quality of life and structural outcomes in the Early Rheumatoid Arthritis Study and Network. Rheumatology (Oxford).

[20] Scott IC, Ibrahim F, Panayi G (2019). The frequency of remission and low disease activity in patients with rheumatoid arthritis, and their ability to identify people with low disability and normal quality of life. Semin Arthritis Rheum.

[21] Aletaha D, Landewe R, Karonitsch T (2008). Reporting disease activity in clinical trials of patients with rheumatoid arthritis: EULAR/ACR collaborative recommendations. Arthritis Rheum.

[22] Smolen JS, Breedveld FC, Burmester GR (2016). Treating rheumatoid arthritis to target: 2014 update of the recommendations of an international task force. Ann Rheum Dis.

[23] Nguyen H, Ruyssen-Witrand A, Gandjbakhch F, Constantin A, Foltz V, Cantagrel A (2014). Prevalence of ultrasound-detected residual synovitis and risk of relapse and structural progression in rheumatoid arthritis patients in clinical remission: a systematic review and meta-analysis. Rheumatology (Oxford).

[24] Terslev L, Brahe CH, Ostergaard M (2021). Using a DAS28-CRP-steered treat-to-target strategy does not eliminate subclinical inflammation as assessed by ultrasonography in rheumatoid arthritis patients in longstanding clinical remission. Arthritis Res Ther.

[25] Combe B, Logeart I, Belkacemi MC (2015). Comparison of the long-term outcome for patients with rheumatoid arthritis with persistent moderate disease activity or disease remission during the first year after diagnosis: data from the ESPOIR cohort. Ann Rheum Dis.

[26] Genitsaridi I, Flouri I, Plexousakis D (2020). Rheumatoid arthritis patients on persistent moderate disease activity on biologics have adverse 5-year outcome compared to persistent low-remission status and represent a heterogeneous group. Arthritis Res Ther.

[27] Arts EE, Fransen J, Den Broeder AA, van Riel P, Popa CD. Low disease activity (DAS28</=3.2) reduces the risk of first cardiovascular event in rheumatoid arthritis: a time-dependent Cox regression analysis in a large cohort study. Ann Rheum Dis. 2017;76(10):1693–9. 10.1136/annrheumdis-2016-210997 28606965

[28] Myasoedova E, Chandran A, Ilhan B (2016). The role of rheumatoid arthritis (RA) flare and cumulative burden of RA severity in the risk of cardiovascular disease. Ann Rheum Dis.

[29] Accortt NA, Lesperance T, Liu M (2018). Impact of sustained remission on the risk of serious infection in patients with rheumatoid arthritis. Arthritis Care Res.

[30] Yun H, Chen L, Roy JA, et al. Rheumatoid arthritis disease activity and hospitalized infection in a large U.S. registry. Arthritis Care Res. 2023;75(8):1639–47. 10.1002/acr.24984 PMC10277216 35866713

[31] Kedra J, Seror R, Dieude P, et al. Lymphoma complicating rheumatoid arthritis: results from a French case-control study. RMD Open. 2021;7(3):e001698. 10.1136/rmdopen-2021-001698 PMC8413949 34470830

