# Supplementary material for: Ten-year radiographic and functional outcomes in rheumatoid arthritis patients in remission compared to patients in low disease activity
Source: Arthritis Res Ther. 2023 Oct 20;25:207. doi: 10.1186/s13075-023-03176-7 (PMC10588022; doi:10.1186/s13075-023-03176-7)
Supplement: Supplementary file 1 — Additional file 1: Supplementary Data S1. Grouping patients according to disease activity state. Supplementary Data S2. Sensitivity analysis. Supplementary Data S3. Multivariate analysis. Supplementary Data. Sensitivity analyses. Supplementary Data S4. When using DAS28-ESR to assess disease activity, comparative analyses showed that patients classified in sustained remission according to DAS28-ESR were females in 70% of cases compared to 88% in the sustained LDA group and 92% in the MDA or HDA groups (p < 0.001) and had shorter disease duration (mean duration in remission group: 6.3 months (SD: 9.0) compared to 8.8 months (SD: 7.5) in the LDA group and 8.4 months (SD: 10.1) in the MDA or HDA groups, p < 0.001) and lower ESR (mean ESR in remission group: 24 (SD: 22) compared to 36 (SD: 28) in the LDA group and 39 (SD: 26) in the MDA or HDA groups, p < 0.001 while CRP levels were comparable across the three groups. Baseline HAQ scores were comparable between patients in sustained remission and LDA and lower than patients in the MDA or HDA groups (mean HAQ in the remission group: 0.86 (SD: 0.61) and 0.88 (SD: 0.64) in the LDA group compared to 1.28 (SD: 0.69) in the MDA or HDA groups, p < 0.001). Patients in the sustained remission group were less exposed to corticosteroids, DMARDs and bDMARDs during the follow-up in the cohort compared to patients in sustained LDA and sustained MDA or HDA (see Table S1). Univariate analyses revealed that patients in sustained remission had lower ten-year mTSS scores and ten-year HAQ scores compared to patients in sustained LDA and patients in sustained MDA or HDA (ten-year mTSS mean in remission group: 47.94 (10.75), compared to 12.18 (16.66) in the LDA group and 22.66 (25.19) in the MDA or HDA groups, p < 0.001, ten-year HAQ in remission group: 0.24 (0.38) compared to 0.58 (0.49) in the LDA group and 1.21 (0.68), p < 0.001). Figure S1. Profile with 95% confidence intervals of each group of patients provided by the optimal lcmm model. [file 13075_2023_3176_MOESM1_ESM.docx]

**Supplementary material**

**Methods**

Supplementary data S1: Grouping patients according to disease activity state

Several methods have been tested to guarantee optimal clusters of patients according to their disease activity state. These groups had to be large enough to allow the comparative studies to be carried out a posteriori.

Method 1: Built clusters of patients according to their levels of disease activity at each visit within the ten-year follow-up.
We first considered grouping patients according to their level of disease activity between the first one-year visit and the ten-year visit using the validated remission and LDA thresholds of SDAI and DAS28-ESR. This method only provided seven patients in sustained remission according to the SDAI and 21 patients according to the DAS28-ESR score. Moreover, we identified with this method only one patient in SDAI-LDA and one patient in DAS28-ESR-LDA. Due to an obvious lack of cases and an important loss of information between visits, this method was quickly abandoned.

Method 2: Grouping patients according to their disease activity level trajectory.

We proposed an optimal longitudinal latent class mixed model (called “lcmm”, [1, 2] ) to identify homogeneous trajectories of disease activity using the course of the SDAI score as the outcome. We conducted several models by varying the number of clusters (between 2 and 4), the link function of the model (linear, beta or spline function) clusters, with or without missing data imputation. Multiple imputations were possibly used here because the proportion of incomplete information was limited. The predictions of the proposed models obtained are presented below. All these models were implemented via the lcmm package of the R software [3] and multiple imputations were carried out using the MICE package [4].

One of the models provided the minimal Bayesian information criterion(BIC, [5]) and so appeared to be optimal. It identified four distinct patient trajectories: a patient trajectory that appears to be in prolonged remission, two patient trajectories with low activity and a patient trajectory with moderate to high activity. The distribution of activity scores by time for each group with this model is presented below.


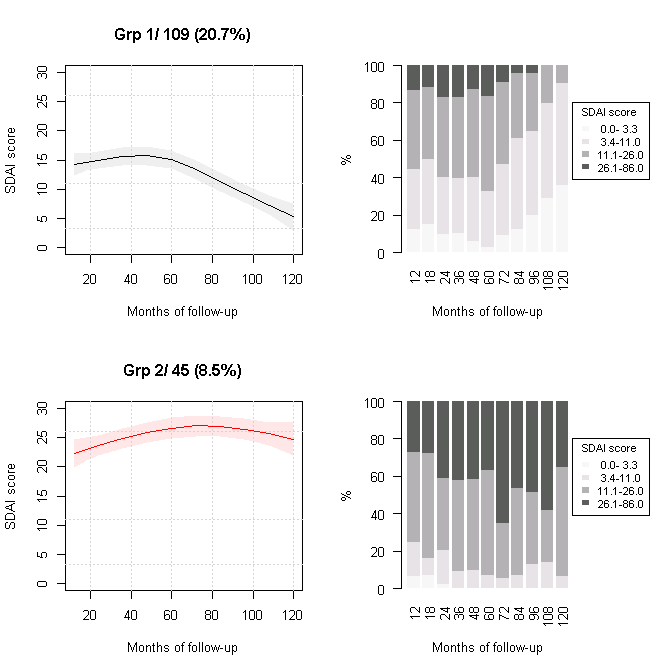


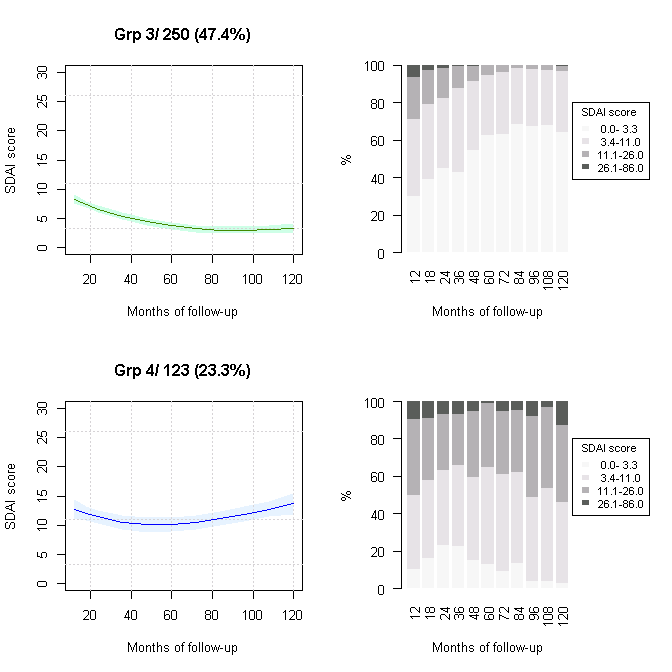


Figure S1: Profile with 95% confidence intervals of each group of patients provided by the optimal lcmm model. The right column provides the composition of each cluster in time by levels of SDAI score. (‘/number’ corresponds to the number of patients by cluster)

Since group 1 and group 4 seemed to have trajectories in LDA boundaries, we decided to group the patients of these two groups. We then verified the individual patient data according to their identified groups. Nevertheless, after manual verification, we found that this method of patient clustering did not adequately separate patients in sustained remission from patients in sustained LDA (examples of individual patient trajectories below) and so provided unsatisfactory results.

Supplementary Table S1: Example of SDAI

| Patient | Group | V2 | V3 | V4 | V5 | V6 | V7 | V8 | V9 | V10 | V11 | V12 |
| --- | --- | --- | --- | --- | --- | --- | --- | --- | --- | --- | --- | --- |
| 12/021 | LDA | 12.40 | 9.30 | 5.80 | 10.50 | 4.50 | 1.30 | 3.10 | 4.10 | 3.30 | 4.86 | 1.50 |
| 10/008 | REM | 1.80 | 5.90 | 5.50 | 3.70 | 4.10 | 5.28 | 2.70 | 4.10 | 6.10 | 1.00 | 1.50 |
| 03/004 | LDA | 0.8 | 6.4 | 25.9 | 17.1 | 0.9 | 2.6 | 4.4 | 2.2 | 2.8 | 3.1 | 4.5 |
| 04/033 | LDA | 1.6 | 0.3 | 1.3 | 11.6 | 3 | 0.6 | 0.6 | 0.2 | 0.1 | 0.9 | 0.1 |

Indeed, when considering patient 10/008, classified in the remission group, we noted that in 6 visits of 11, he/she had an SDAI>3.3. In contrast, patient 04/033, classified in the LDA group, had 10 visits of 11 with an SDAI<3.3 and no visit with an SDAI in LDA.

Method 3: definition of SDAI and DAS28 states with predefined rules.

After the study, we concluded that methods 1 and 2 did not allow for properly separating patients in sustained LDA from patients in sustained remission. Therefore, we sought to group patients according to their level of activity by allowing moderate activity fluctuations. The objective was to define sufficiently stringent boundaries for the patient to be representative of their activity level group but also to obtain enough patients per group to allow comparative analyses. By re-sampling the raw database (bootstrap [6], B = 100 replicates), we were able to define bootstrapped tertiles within the validated threshold range for disease activity scores. These tertiles are described for the SDAI and DAS28-ESR, respectively, in the table below. They can be considered as acceptable confidence zones for a patient to be classed in their correct disease activity state by the standard classification. The first tertile of the next disease activity state was used to define the new accepted threshold. For example, the first tertile of SDAI LDA was 5.48. Thus, this value was used to define a new accepted threshold for SDAI remission status in a limited number of visits.

Table S2: Details of the bootstrapped tertiles including validity thresholds (standard method) by disease activity states for SDAI (a) and DAS28 (b). HDA: high disease activity, MDA: moderate disease activity, LDA: low disease activity.

| **Disease activity state** | **Thresholds** | **Values** |
| --- | --- | --- |
| HDA | 2nd tertile | **6.46** |
| HDA | 1st tertile | 5.85 |
| MDA | **Validated** | **5.1** |
| MDA | 2nd tertile | 4.42 |
| MDA | 1st tertile | 3.78 |
| LDA | **Validated** | **3.20** |
| LDA | 2nd tertile | 3.00 |
| LDA | 1st tertile | 2.80 |
| R | **Validated** | **2.60** |
| R | 2nd tertile | 2.22 |
| R | 1st tertile | 1.76 |
| R | **0.0** | **0.00** |

1. *DAS28 score*

| **Disease activity state** | **Thresholds** | **Values** |
| --- | --- | --- |
| HDA | 2nd tertile | **40.49** |
| HDA | 1st tertile | 32.69 |
| MDA | **Validated** | **26.00** |
| MDA | 2nd tertile | 19.14 |
| MDA | 1st tertile | 14.84 |
| LDA | **Validated** | **11.00** |
| LDA | 2nd tertile | 8.30 |
| LDA | 1st tertile | 5.48 |
| R | **Validated** | **3.30** |
| R | 2nd tertile | 2.15 |
| R | 1st tertile | 0.97 |
| R | **0.0** | **0.00** |

1. *SDAI score*

Once these limits were defined, the following rules were applied to constitute the groups.
The sustained remission group was defined if patients had:
- At least 70% of the visits with an SDAI in the 0 – 3.3 range
- At least 90% of visits with an SDAI in the 0 – 5.48 range, which is the value of the first upper tertile of the LDA status.
- And no visits with SDAI >11.

Group 2 with sustained LDA was defined as follows:
- At least 90% of the visits with an SDAI in the 0.97-19.14 range (between the second tertile of remission status and the first upper tertile of MDA or HDA status)
- And a median of the effective measures in the 3.3 – 11 range, in order to avoid excessive dispersion of the activity measures

Group 3 patients with moderate to high activity were defined as having:
- At least 60% of visits with SDAI >11
- And at least 80% of visits with an SDAI above 8.3 being the second tertile of LDA status
- At least 90% of the SDAI>3.3.

Using these rules, 48 patients were classified in the sustained remission group, 139 patients in the sustained LDA group and 69 patients in the MDA or HDA groups.

The same rules were applied for DAS28:

The sustained remission group was defined if patients had:
- At least 70% of the visits with a DAS28 in the 0 – 2.6 range
- At least 90% of visits with a DAS28 in the 0 – 2.8 range, which is the value of the first upper tertile of the LDA status.
- And no visits with DAS28 >3.2.

Group 2 with sustained LDA was defined as follows:
- At least 90% of the visits with a DAS28 in the 1.76-4.42 range (between the second tertile of remission status and the first upper tertile of MDA or HDA status)
- And a median of the effective measures in the 2.6-3.2 range, in order to avoid excessive dispersion of the activity measures

Group 3 patients with moderate to high activity were defined as having:
- At least 60% of visits with a DAS28 >3.2
- And at least 80% of visits with a DAS28 above 3.0 being the second tertile of LDA status
- At least 90% of the DAS28>2.6.

Using these rules, 79 patients were classified in the sustained remission group, 53 patients in the sustained LDA group and 71 patients in the MDA or HDA group.

Supplementary figure S2: Individual patient trajectory profiles are represented in the graphs below, according to different groups. SDAI trajectories in the whole cohort and in the three groups (group 1= remission, group 2 = LDA, group 3 = MDA or HDA group)

**
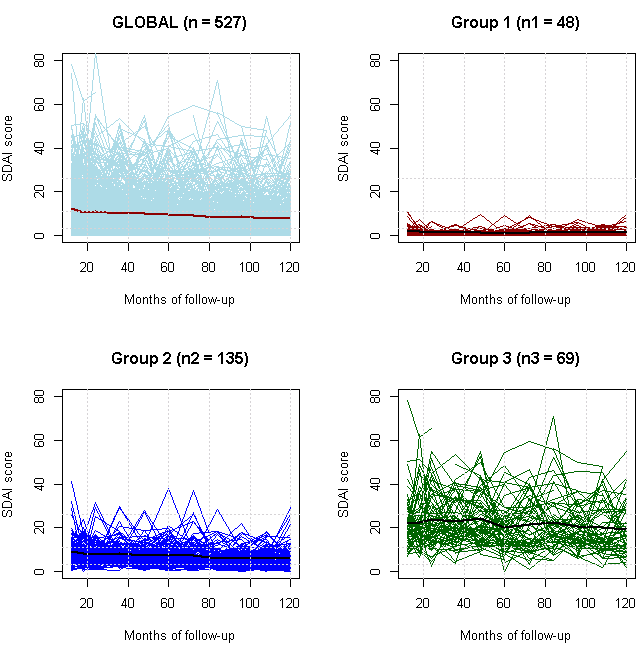
**

Supplementary figure S3: DAS28 trajectories in the whole cohort and in the three groups (group 1 = remission, group 2 = LDA, group 3 = MDA or HDA group)


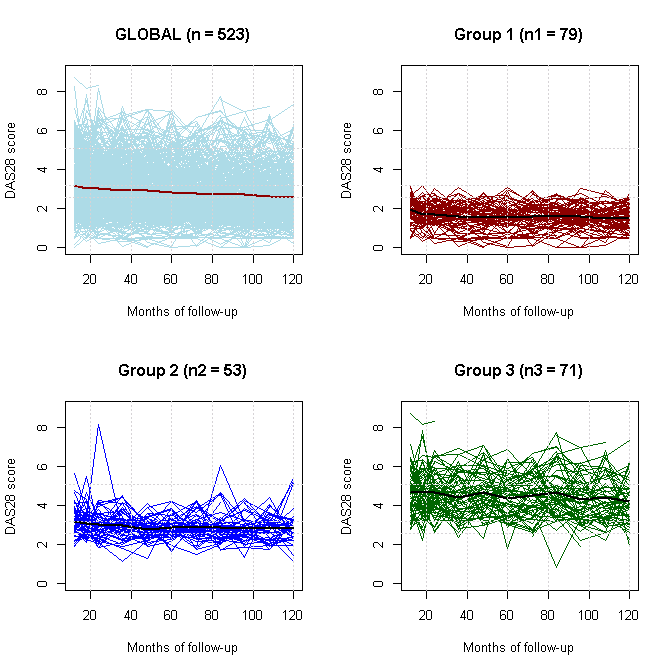


Supplementary data S2: Sensitivity analysis

In order to verify the robustness of the results, we performed a sensitivity analysis by varying the thresholds that had been defined previously. The different rules tested and the number of patients thus identified are detailed in the table below.

Table S3: SDAI group definition sensitivity analysis

| Disease activity status | First pre-defined rules | Sensitivity analyses |
| --- | --- | --- |
| Sustained remission | - At least 70% of the visits with an SDAI in the 0 – 3.3 range - at least 90% of visits with an SDAI in the 0 – 5.48 range - And no visits with an SDAI >11. | **Sensitivity analysis 1a:**  - At least 70% of the visits with an SDAI in the 0 – 3.3 range  - And no visits with SDAI >11.  (the second condition was removed)  **Sensitivity analysis 1b:**  - At least 70% of the visits with an SDAI in the 0 – 3.3 range - **At least 80**% of visits with an SDAI in the 0 – 5.48 range - And no visits with SDAI >11.  **Sensitivity Analysis 1c:**  - **At least 60%** of the visits with an SDAI in the 0 – 3.3 range - At least 90% of visits with an SDAI in the 0 – 5.48 range - And no visits with SDAI >11.  **Sensitivity analysis 1d:**  - **At least 60%** of the visits with an SDAI in the 0 – 3.3 range - **At least 80%** of visits with an SDAI in the 0 – 5.48 range - And no visits with SDAI >11.  **Sensitivity analysis 1e:**  - At least 70% of the visits with an SDAI in the 0 – 3.3 range - At least 90% of visits with an SDAI in the **0 – 8.30** range (second tertile of the LDA status) - And no visits with SDAI >11. |
| Sustained LDA | - At least 90% of the visits with an SDAI in the 0.97-19.14 range - And a median of the effective measures in the 3.3 – 11 range | **Sensitivity analysis 2a:**  - At least 90% of the visits with an SDAI in the 0.97-19.14 range  (the second condition was removed)  **Sensitivity analysis 2b:**  - A median of the effective measures in the 3.3 – 11 range  (the first condition was removed)  **Sensitivity analysis 2c:**  - **At least 80%** of the visits with an SDAI in the 0.97-19.14 range - And a median of the effective measures in the 3.3 – 11 range  **Sensitivity analysis 2d:**  - At least 90% of the visits with an SDAI in the 0.97-19.14 range - And a median of the effective measures in the **2.15 – 14.84** range (second tertiles) |
| Sustained MDA or HDA | - At least 60% of visits with SDAI >11 - And at least 80% of visits with an SDAI above 8.3  - At least 90% of the SDAI>3.3 | **Sensitivity analysis 3a:**  - And at least 80% of visits with an SDAI above 8.3  - At least 90% of the SDAI>3.3  (the first condition was removed)  **Sensitivity analysis 3b:**  - At least 60% of visits with SDAI >11 - At least 90% of the SDAI>3.3  (the second condition was removed)  **Sensitivity analysis 3c:**  - At least 60% of visits with SDAI >11 - And at least 80% of visits with an SDAI above 8.3  (the third condition was removed)  **Sensitivity analysis 3d:**  - At least 60% of visits with SDAI >11 - And at least **70%** of visits with an SDAI above 8.3  - At least 90% of the SDAI>3.3  **Sensitivity analysis 3e:**  - At least 60% of visits with SDAI >11 - And at least 80% of visits with an SDAI above 8.3  - At least 90% of the SDAI>2.15 (second tertile of remission status) |

Table S4: DAS28-ESR group definition sensitivity analysis

| Disease activity status | First pre-defined rules | Sensitivity analyses |
| --- | --- | --- |
| Sustained remission | - At least 70% of the visits with a DAS28 in the 0 – 2.6 range - At least 90% of visits with a DAS28 in the 0 – 2.8 range - And no visits with a DAS28 >3.2. | **Sensitivity analysis 1a:**  - At least 70% of the visits with a DAS28 in the 0 – 2.6 range  - And no visits with DAS28 >3.2.  (the second condition was removed)  **Sensitivity analysis 1b:**  - At least 70% of the visits with a DAS28 in the 0 – 2.6 range - **At least 80**% of visits with a DAS28 in the 0 – 2.8 range - And no visits with DAS28 >3.2.  **Sensitivity Analysis 1c:**  - **At least 60%** of the visits with a DAS28 in the 0 – 2.6 range - At least 90% of visits with a DAS28 in the 0 – 2.8 range - And no visits with a DAS28 >3.2.  **Sensitivity analysis 1d:**  - **At least 60%** of the visits with a DAS28 in the 0 – 2.6 range - **At least 80%** of visits with a DAS28 in the 0 – 2.8 range  - And no visits with a DAS28 >3.2.  **Sensitivity analysis 1e:**  - At least 70% of the visits with a DAS28 in the 0 – 2.6 range - At least 90% of visits with a DAS28 in the **0 – 3.0** range (second tertile of the LDA status) - And no visits with a DAS28 >3.2. |
| Sustained LDA | - At least 90% of the visits with a DAS28 in the 1.76-4.42 range  - And a median of the effective measures in the 2.6 – 3.2 range | **Sensitivity analysis 2a:**  - At least 90% of the visits with a DAS28 in the 1.76-4.42 range  (the second condition was removed)  **Sensitivity analysis 2b:**  - A median of the effective measures in the 2.6 – 3.2 range  (the first condition was removed)  **Sensitivity analysis 2c:**  - **At least 80%** of the visits with a DAS28 in the 1.76-4.42 range  - And a median of the effective measures in the 2.6 – 3.2 range  **Sensitivity analysis 2d:**  - At least 90% of the visits with a DAS28 in the 0.97-19.14 range - And a median of the effective measures in the **2.22 – 3.78** range (second tertiles) |
| Sustained MDA or HDA | - At least 60% of visits with a DAS28 >3.2 - And at least 80% of visits with a DAS28 above 3.0 - At least 90% of the DAS28>2.6 | **Sensitivity analysis 3a:**  - And at least 80% of visits with a DAS28 above 3.0  - At least 90% of the DAS28>2.6  (the first condition was removed)  **Sensitivity analysis 3b:**  - At least 60% of visits with a DAS28 >3.2 - At least 90% of the DAS28>2.6  (the second condition was removed)  **Sensitivity analysis 3c:**  - At least 60% of visits with a DAS28 >3.2 - And at least 80% of visits with a DAS28 above 3.0  (the third condition was removed)  **Sensitivity analysis 3d:**  - At least 60% of visits with a DAS28 >3.2 - And at least **70%** of visits with a DAS28 above 3.0  - At least 90% of the DAS28>2.6  **Sensitivity analysis 3e:**  - At least 60% of visits with a DAS28 >3.2 - And at least 80% of visits with a DAS28 above 3.0  - At least 90% of the DAS28>2.22 (second tertile of remission status) |

Supplementary data S3: Multivariate analysis

Model to predict mTSS progression

To describe, as far as possible, the changes over time in measurement scales (and notably consider their distributional properties at best), we used latent process mixed models [3].

Five structural measures of mTSS were available in the ESPOIR cohort and considered in a unique final model: inclusion, two-year, five-year, seven-year and ten-year visits. The prediction of mTSS suggested using quadratic I-splines with five knots [7] as an optimal link function of the model. The best model structure without covariate adjustments initially kept: a quadratic function of time and its interaction with the DAS28-ESR previously constructed as fixed effects while the centre of inclusion corresponded to the unique random effect. In a second time of study, covariate adjustments were added via a forward selection process comparing the p-values of global Wald tests (with a p-value<0.05 as the significance threshold). The final model included all the variables associated with a p-value <0.05. 59 patients with no radiographic measures were excluded from this analysis.

The list of potential covariate adjustments was age, gender, smoking, ACPA presence, rheumatoid factor presence, typical erosions, disease duration, CRP, ESR, corticosteroids and DMARD (including csDMARD and bDMARD) exposure (exposure as binary outcome and exposure duration for each drug).

Slope differences over the entire duration of follow-up (10 years), and mean differences after 10 years between groups of DAS28-ESR were estimated by testing appropriate linear combinations from the final model parameters [8]. The Wald tests related to these contrasts were implemented in R software via the WaldMult function of the lcmm package.

Model to predict ten-year HAQ

The same approach was used for the longitudinal model to predict the ten-year HAQ. All values of HAQ available at each visit were used. The optimal model corresponded to a time quadratic model with an I-spline (three knots) link function.

Cox model to predict ten-year serious adverse events risk

A binary outcome called “serious adverse events” has been constructed from information collected on patients over the ten years of follow-up: it noted, for each patient, the presence (encoded 1) or absence (encoded 0) of at least one of the following pathologies during each of its monitoring visits: serious infections, neoplasia, major cardiovascular events (MACE), thromboembolism events, or death.

From a sample composed of 203 patients that corresponded to the 203 patients previously classified in one of the three DAS28-ESR groups (REM: 79, LDA: 53, MDA or HDA: 71), a Cox model [9] has been used to estimate the effect of rheumatoid arthritis activity on the risk of serious adverse events during the ten years of follow-up. The exhaustive list of covariate adjustments considered for inclusion in the model was age, sex, disease duration, smoking status, ACPA presence, typical erosions at baseline, smoking status, body mass index, DMARD exposure, corticosteroid exposure, HAQ, Total Sharp Socre at baseline, history of serious infection, history of cancer, history of MACE, history of thromboembolism event, high blood pressure, dyslipidaemia, diabetes.

Among them, time-dependent covariates have been considered in the model by simply encoded intervals of time (inclusion of several lines per patient in the database) [10]. Covariates that did not respect the log-linear and/or proportional hazard assumptions were all recoded before their inclusion in the Cox model.

Starting from a univariate Cox model (stratified by inclusion centre) with the DAS28-ESR as unique factor, covariate adjustments were included in the final model using a bidirectional selection process (the Wald test p-value was the selection criterion) [11]. The analyses were made using the function of the survival package from R software [12] (R Core Team, 2022).

Finally, a similar statistical approach has been used with the 252 patients of the three SDAI-ESR groups (REM: 48, LDA: 135, MDA or HDA: 69), to understand the effect of rheumatoid arthritis activity state (assessed by SDAI-ESR) on the risk of serious adverse events during the ten years of follow-up.

**Results**

Table S5: Inclusion patient characteristics and treatments taken over time by their DAS28-ESR group during the ten-year follow-up in the ESPOIR cohort.

| **Patient characteristics** | **REM**  **N=79** | **LDA**  **N=53** | **MDA or HDA**  **N=71** | **P-value** |
| --- | --- | --- | --- | --- |
| **Baseline characteristics** |  |  |  |  |
| Age, years, mean (SD) | 47.2 (12.4) | 51.4 (9.0) | 50.2 (10.9) | NS |
| Number of female sujects (%) | 55 (70) | 46 (88) | 65 (92) | 0.001 |
| Disease duration, months, mean (SD) | 6.3 (9.0) | 8.8 (7.5) | 8.4 (10.1) | 0.001 |
| RF, number (%) | 34 (43) | 30 (57) | 34 (48) | NS |
| ACPA, number (%) | 31 (39) | 29 (55) | 32 (45) | NS |
| Typical erosions, number of patients (%) | 32 (41) | 28 (53) | 40 (56) | NS |
| ESR, mean (SD) | 24.0 (22.4) | 36.0 (27.9) | 38.8 (26.2) | <0.001 |
| CRP mean (SD) | 22.9 (29.9) | 20.3 (25.5) | 25.3 (36.4) | NS |
| mTSharp, mean (SD) | 2.40 (4.66) | 2.43 (4.02) | 4.53 (7.79) | NS |
| HAQ, mean (SD) | 0.86 (0.61) | 0.88 (0.64) | 1.28 (0.69) | < 0.001 |
| Smokers, number (%) | 37 (47%) | 21(40%) | 30 (42%) | NS |
| **Ten-year characteristics** |  | | | |
| Corticosteroids, number of patients (%) | 53 (67) | 45 (85) | 65 (92) | <0.001 |
| Corticosteroid cumulative dose, gr, mean (SD) | 1.9 (4.1) | 4.4 (6.9) | 7.0 (11.0) | < 0.001 |
| DMARDs, number of patients (%) | 62 (78) | 48 (90) | 66 (92) | 0.05 |
| DMARDs exposure duration, months, mean (SD) | 62.1 (50.4) | 86.6 (46.1) | 83.5 (43.0) | 0.01 |
| Methotrexate, number of patients (%) | 53 (67) | 40 (75) | 62 (87) | 0.01 |
| Methotrexate exposure duration, months, mean (SD) | 57.2 (51.9) | 67.9 (53.3) | 69.4 (48.0) | NS |
| bDMARDs, number of patients (%) | 11 (14) | 18 (34) | 37 (52) | < 0.001 |
| bDMARDs exposure duration, months, mean (SD) | 9.3 (26.8) | 16.6 (29.3) | 31.8 (41.1) | < 0.001 |
| Ten-year mTSS, mean (SD) | 7.94 (10.75) | 12.18 (16.66) | 22.66 (25.19) | < 0.001 |
| Ten-year HAQ, mean (SD) | 0.24 (0.38) | 0.58 (0.49) | 1.21 (0.68) | < 0.001 |

REM: patients in sustained remission, LDA: patients in sustained low disease activity, MDA: patients in moderate disease activity, HDA: patients in sustained high disease activity; SD: Standard deviation, RF: rheumatoid factor, ACPA: anti-citrullinated peptides antibodies, mTSS: Van der Heijde modified Total Sharpe Score, HAQ: Health Assessment Questionnaire, DMARDs: Disease Modifying Anti-Rheumatic Drugs, bDMARDs: biologic Disease Modifying Anti-Rheumatic Drugs

**Supplementary data S4**: When using DAS28-ESR to assess disease activity, comparative analyses showed that patients classified in sustained remission according to DAS28-ESR were females in 70% of cases compared to 88% in the sustained LDA group and 92% in the MDA or HDA groups (p<0.001) and had shorter disease duration (mean duration in remission group: 6.3 months (SD: 9.0) compared to 8.8 months (SD: 7.5) in the LDA group and 8.4 months (SD: 10.1) in the MDA or HDA groups, p<0.001) and lower ESR (mean ESR in remission group: 24 (SD: 22) compared to 36 (SD: 28) in the LDA group and 39 (SD: 26) in the MDA or HDA groups, p<0.001 while CRP levels were comparable across the three groups. Baseline HAQ scores were comparable between patients in sustained remission and LDA and lower than patients in the MDA or HDA groups (mean HAQ in the remission group: 0.86 (SD: 0.61) and 0.88 (SD: 0.64) in the LDA group compared to 1.28 (SD: 0.69) in the MDA or HDA groups, p<0.001). Patients in the sustained remission group were less exposed to corticosteroids, DMARDs and bDMARDs during the follow-up in the cohort compared to patients in sustained LDA and sustained MDA or HDA (see table S1). Univariate analyses revealed that patients in sustained remission had lower ten-year mTSS scores and ten-year HAQ scores compared to patients in sustained LDA and patients in sustained MDA or HDA (ten-year mTSS mean in remission group: 47.94 (10.75), compared to 12.18 (16.66) in the LDA group and 22.66 (25.19) in the MDA or HDA groups, p<0.001, ten-year HAQ in remission group: 0.24 (0.38) compared to 0.58 (0.49) in the LDA group and 1.21 (0.68), p<0.001).

**Association between disease activity groups and variation of mTSS within ten years (Supplementary figure S6, supplementary Table S7).**

Supplementary Figure S6: Profiles (with 95% confidence intervals) of mTSS trajectories over ten years using the DAS28-ESR score for disease activity-based groups

**
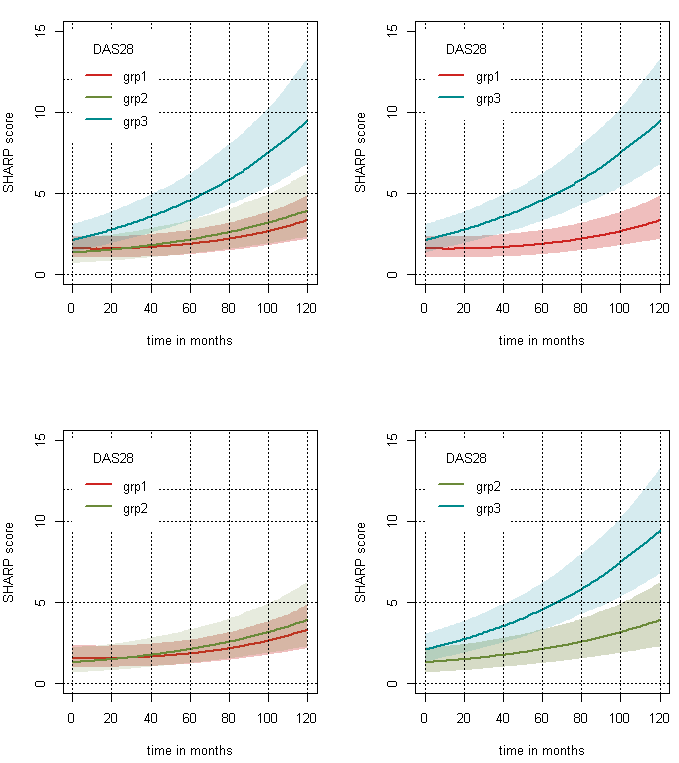
**

Group 1: sustained remission group, group 2: sustained LDA group, group 3: sustained MDA or HDA group

Table S7 : Multivariate analysis assessing the ten-year mTSS progression and ten-year HAQ using DAS28-ESR as disease activity score: Wald tests related to the parameter estimates and contrast of the final model

| Outcome | Variables | Wald test | p value |
| --- | --- | --- | --- |
| Ten-year mTSS progression | Baseline erosions | 46.9 | <0.0001 |
|  | DAS28-ESR group | 2.9 | NS |
|  | DAS28-ESR group* time | 16.1 | <0.0001 |
|  | DAS28-ESR*intercept^2^ | 2.5 | NS |
|  | ACPA | 0.7 | NS |
|  | ACPA*time | 39.8 | <0.0001 |
|  | ACPA *intercept^2^ | 13.2 | <0.001 |
|  | Time | 0.4 | NS |
|  | Intercept^2^ | 4.3 | <0.05 |
| Results of the contrast method of ten-year mTSS progression | Final model:  LDA versus remission  MDA or HDA versus remission  MDA or HDA versus LDA | 1.5  5.2  3.2 | NS  <0.0001  <0.01 |
| Ten-year HAQ | RF | 4.8 | <0.05 |
|  | bDMARD | 5.2 | <0.05 |
|  | DAS28 group | 54.7 | <0.0001 |
|  | DAS28 group*time | 79.7 | <0.0001 |
|  | DAS28 group*intercept^2^ | 60.2 | <0.0001 |
|  | Methotrexate use | 0.1 | NS |
|  | Methotrexate*time | 11.6 | <0.001 |
|  | Methotrexate*intercept^2^ | 8.09 | <0.01 |
|  | Corticosteroids use | 2.0 | NS |
|  | Corticosteroids use*time | 5.5 | <0.05 |
|  | Corticosteroids use*intercept^2^ | 8.1 | <0.01 |
|  | Baseline erosions | 0.3 | NS |
|  | Baseline erosions*time | 6.6 | <0.05 |
|  | Baseline erosions*intercept^2^ | 4.8 | NS |
|  | Time | 4.7 | <0.05 |
|  | Intercept^2^ | 2.3 | NS |
| Results of the contrast method of ten-year HAQ | Final model:  LDA versus remission  MDA or HDA versus remission  MDA or HDA versus LDA | 3.4  8.5  4.8 | <0.001  <0.0001  <0.0001 |

mTSS: van der Heijde modified Total Sharp Score, SDAI: Simple Disease Activity Index, ACPA: anti-citrullinated peptide antibodies, DMARD: Disease Modifying Anti-Rheumatic Drugs, bDMARDs: biologic Disease Modifying Anti-Rheumatic Drugs. The included variables tested with univariate then multivariate analyses included: disease activity group based on DAS28-ESR, age, sex, smoking, duration of symptoms, centre of inclusion, presence of rheumatoid and/or ACPA factors, presence of erosion at diagnosis, synthetic background treatment, corticosteroids, bDMARD use

**Association between disease activity groups and ten-year HAQ (Supplementary Figure S8, supplementary Table S7).**

Supplementary Figure S8: Results of the modelisation of HAQ evolution over ten years using the DAS28-ESR score for disease activity-based groups


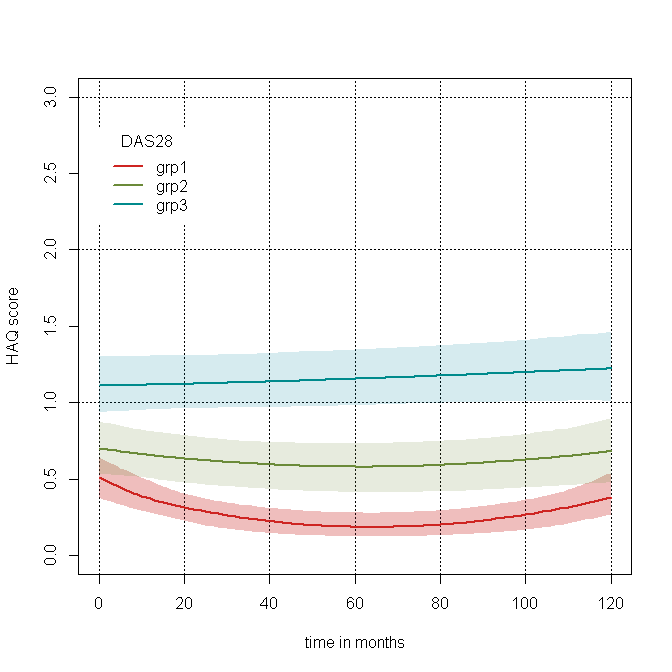


**Supplementary data: Sensitivity analyses**

To verify the consistency of the results, we performed a sensitivity analysis changing the threshold definition of the three SDAI and DAS28-ESR groups. Globally, after changing the definition of the groups, the results remained consistent with the primary analysis.

Sensitivity analyses with SDAI:

Table S9: Sensitivity analysis by modifying the rules of the definition of the three groups based on SDAI as disease activity score. Comparison of ten-year mTSS progression across the three groups using contrasts from the final model parameters

| Sensitivity analysis | LDA versus remission  Wald test, p-value | MDA or HDA versus remission  Wald test, p-value | MDA or HDA versus LDA, p-value |
| --- | --- | --- | --- |
| Reference model | 3.2, p<0.01 | 4.5, p<0.0001 | 2.1, p<0.05 |
| Sensitivity analysis 1a  Sensitivity analysis 1b  Sensitivity analysis 1c  Sensitivity analysis 1d  Sensitivity analysis 1e | 3.0, p<0.05  3.0, p<0.01  3.2, p<0.01  3.2, p<0.01  2.9, p<0.01 | 4.4, p<0.0001  4.4, p<0.0001  4.6, p<0.0001  4.6, p<0.0001  4.3, p<0.0001 | 2.1, p<0.05  2.1, p<0.05  2.1, p<0.05  2.0, p<0.05  2.1, p<0.05 |
| Sensitivity analysis 2a  Sensitivity analysis 2b  Sensitivity analysis 2c  Sensitivity analysis 2d | 3.4, p<0.001  3.4, p<0.001  3.3, p<0.001  3.2, p<0.01 | 4.6, p<0.0001  4.3, p<0.0001  4.4, p<0.0001  4.7, p<0.0001 | 2.2, p<0.05  2.0, p<0.05  2.0, p<0.05  1.9, NS |
| Sensitivity analysis 3a  Sensitivity analysis 3b  Sensitivity analysis 3c  Sensitivity analysis 3d  Sensitivity analysis 3e | 3.1, p<0.01  3.0, p<0.01  3.2, p<0.01  3.0, p<0.01  3.2, p<0.01 | 4.8, p<0.0001  5.1, p<0.0001  4.7, p<0.0001  5.0, p<0.0001  4.5, p<0.0001 | 2.5, p<0.05  3.0, p<0.01  2.4, p<0.05  2.9, p<0.01  2.1, p<0.05 |

Table S10: Sensitivity analysis by modifying the rules of the definition of the three groups based on SDAI as disease activity score. Comparison of ten-year HAQ across the three groups using contrasts from the final model parameters.

| Sensitivity analysis | LDA versus remission  Wald test, p-value | MDA or HDA versus remission  Wald test, p-value | MDA or HDA versus LDA, p-value |
| --- | --- | --- | --- |
| Reference model | 3.5, p<0.001 | 4.6, p<0.0001 | 2.0, p<0.05 |
| Sensitivity analysis 1a  Sensitivity analysis 1b  Sensitivity analysis 1c  Sensitivity analysis 1d  Sensitivity analysis 1e | 3.2, p<0.01  3.3, p<0.001  3.5, p<0.01  3.5, p<0.001  3.1, p<0.01 | 4.4, p<0.0001  4.5, p<0.0001  4.7, p<0.0001  4.7, p<0.0001  4.3, p<0.0001 | 1.9, p<0.05  2.0, p<0.05  2.0, p<0.05  2.0, p<0.05  2.0, p<0.05 |
| Sensitivity analysis 2a  Sensitivity analysis 2b  Sensitivity analysis 2c  Sensitivity analysis 2d | 3.4, p<0.001  2.6, p<0.01  3.2, p<0.01  4.5, p<0.0001 | 4.5, p<0.0001  4.5, p<0.0001  4.7, p<0.0001  4.8, p<0.0001 | 2.1, p<0.05  3.2, p<0.01  2.6, p<0.01  0.8, NS |
| Sensitivity analysis 3a  Sensitivity analysis 3b  Sensitivity analysis 3c  Sensitivity analysis 3d  Sensitivity analysis 3e | 3.4, p<0.001  3.3, p<0.001  3.5, p<0.01  3.3, p<0.001  3.5, p<0.001 | 4.6, p<0.0001  4.0, p<0.0001  4.8, p<0.0001  4.2, p<0.0001  4.7, p<0.0001 | 2.1, p<0.05  1.2, NS  2.2, p<0.05  1.4, NS  2.1, p<0.05 |

Sensitivity analyses with DAS28-ESR

Globally, after changing the definition of the groups, the results remained consistent with the primary analysis, except for the comparison between patients in sustained DAS28-ESR remission and LDA on mTSS progression with marginal significant association when slightly changing the definition of patients in sustained LDA (see table S12 and S13).

Table S11: Sensitivity analysis by modifying the rules of the definition of the three groups based on DAS28-ESR as the disease activity score. Comparison of ten-year mTSS progression across the three groups using contrasts from the final model parameters.

| Sensitivity analysis | LDA versus remission  Wald test, p-value | MDA or HDA versus remission  Wald test, p-value | MDA or HDA versus LDA, p-value |
| --- | --- | --- | --- |
| Reference model | 1.5, NS | 5.2, p<0.0001 | 3.2, p<0.01 |
| Sensitivity analysis 1a  Sensitivity analysis 1b  Sensitivity analysis 1c  Sensitivity analysis 1d  Sensitivity analysis 1e | 1.7, NS  1.8, NS  1.6, NS  1.9, NS  1.6, NS | 5.4, p<0.0001  5.5, p<0.0001  5.3, p<0.0001  5.6, p<0.0001  5.3, p<0.0001 | 3.2, p<0.01  3.1, p<0.01  3.2, p<0.01  3.1, p<0.01  3.2, p<0.01 |
| Sensitivity analysis 2a  Sensitivity analysis 2b  Sensitivity analysis 2c  Sensitivity analysis 2d | 2.4, p<0.05  2.3, p<0.05  2.2, p<0.05  0.6, NS | 4.5, p<0.0001  4.9, p<0.0001  5.1, p<0.0001  5.1, p<0.0001 | 2.6, p<0.01  2.8, p<0.01  2.8, p<0.01  2.8, p<0.01 |
| Sensitivity analysis 3a  Sensitivity analysis 3b  Sensitivity analysis 3c  Sensitivity analysis 3d  Sensitivity analysis 3e | 1.5, NS  1.5, NS  1.5, NS  1.4, NS  1.5, NS | 5.2, p<0.0001  5.1, p<0.0001  5.5, p<0.0001  5.2, p<0.0001  5.2, p<0.0001 | 3.2, p<0.01  3.2, p<0.01  3.5, p<0.001  3.2, p<0.01  3.2, p<0.01 |

Table S12: Sensitivity analysis by modifying the rules of the definition of the three groups based on DAS28-ESR as the disease activity score. Comparison of ten-year HAQ across the three groups using contrasts from the final model parameters.

| Sensitivity analysis | LDA versus remission  Wald test, p-value | MDA or HDA versus remission  Wald test, p-value | MDA or HDA versus LDA, p-value |
| --- | --- | --- | --- |
| Reference model | 3.4, p<0.001 | 8.5, p<0.0001 | 4.8, p<0.0001 |
| Sensitivity analysis 1a  Sensitivity analysis 1b  Sensitivity analysis 1c  Sensitivity analysis 1d  Sensitivity analysis 1e | 3.3, p<0.001  3.4, p<0.001  3.4, p<0.001  3.5, p<0.001  3.3, p<0.01 | 8.7, p<0.0001  8.9, p<0.0001  8.6, p<0.0001  9.0, p<0.0001  8.6, p<0.0001 | 4.8, p<0.0001  4.8, p<0.0001  4.9, p<0.0001  4.8, p<0.0001  4.8, p<0.0001 |
| Sensitivity analysis 2a  Sensitivity analysis 2b  Sensitivity analysis 2c  Sensitivity analysis 2d | 3.0, p<0.01  4.3, p<0.0001  4.0, p<0.01  3.2, p<0.01 | 8.4, p<0.0001  8.6, p<0.0001  8.7, p<0.0001  8.7, p<0.0001 | 6.5, p<0.0001  5.2, p<0.0001  5.1, p<0.0001  3.6, p<0.0001 |
| Sensitivity analysis 3a  Sensitivity analysis 3b  Sensitivity analysis 3c  Sensitivity analysis 3d  Sensitivity analysis 3e | 3.4, p<0.001  3.4, p<0.001  3.3, p<0.001  3.4, p<0.001  3.2, p<0.01 | 8.5, p<0.0001  8.5, p<0.0001  8.0, p<0.0001  8.6, p<0.0001  8.5, p<0.0001 | 4.8, p<0.0001  4.6, p<0.0001  4.1, p<0.0001  4.7, p<0.0001  4.8, p<0.0001 |

Safety

Table S13: Ten-year serious adverse events according to the three groups of DAS28-ESR: ten-year incidence for 100 patient-years

|  | All population  (1685 PY) | REM  (675PY) | LDA  (444PY) | MDA or HDA  (667 PY) |
| --- | --- | --- | --- | --- |
| Serious adverse events | 2.84 | 2.07 | 2.92 | 3.15 |
| Serious infections | 1.13 | 1.03 | 0.68 | 1.35 |
| Neoplasia | 1.07 | 0.089 | 1.80 | 0.60 |
| MACEs | 0.42 | 0.30 | 0.22 | 0.60 |
| Thromboembolism events | 0.35 | 0.15 | 0.23 | 0.60 |
| Death | 0.24 | 0.15 | 0.23 | 0.30 |

REM: patients in sustained remission, LDA: patients in sustained low disease activity, MDA: patients in moderate disease activity, HDA: patients in sustained high disease activity; PY: patient years; MACEs: major cardiovascular events; All comparisons were non-significant.

Table S14: Hazard ratios of optimal Cox models to predict ten-year serious adverse events risk with SDAI (a) or DAS28-ESR (b) as rheumatoid arthritis activity scale (N(a) = 252; N(b) = 203)

| Disease activity score used to define the three groups | Variables | Hazard ratios [95% CI] | p-value |
| --- | --- | --- | --- |
| (a) SDAI | Disease activity   - Sustained remission - Sustained LDA - Sustained MDA or HDA   Age > 50 years  Baseline erosions  bDMARD use  bDMARD number  bDMARD exposure duration | Ref  0.49 [0.23-1.02]  0.52 [0.21-1.30]  1.04 [1.01-1.30]  1.71 [0.99-2.97]  2.32 [0.82-6.51]  1.55 [0.99-2.42]  0.99 [0.98-0.99] | NS  NS  <0.05  NS  NS  NS  <0.05 |
| (b) DAS28-ESR | Disease activity   - Sustained remission - Sustained LDA - Sustained MDA or HDA   Age > 50 years  Baseline ESR  History of serious adverse event  History of HBP  bDMARD number  bDMARD exposure duration | ref  1.09 [0.41-2.91]  2.37 [0.48-3.91]  2.90 [0.96-8.74]  1.02 [1.00-1.04]  0.16 [0.03-0.78]  0.16 [0.03-1.07]  1.9 [1.24-3.09]  0.99 [0.98-0.99] | NS  NS  NS  <0.05  <0.05  NS  <0.001  <0.05 |

References

1. McCulloch CE, Lin H, Slate EH, Turnbull BW. Discovering subpopulation structure with latent class mixed models. Stat Med. 2002 Feb 15; 21(3):417-429.

2. McCutcheon AL. Latent class analysis., 1987.

3. Proust-Lima C, Amieva H, Jacqmin-Gadda H. Analysis of multivariate mixed longitudinal data: A flexible latent process approach, 2012.

4. van Buuren S, Groothuis-Oudshoorn K. mice: Multivariate Imputation by Chained Equations in R. . Journal of Statistical Software. 2011; 45(3):1-67.

5. Schwarz GE. Estimating the dimension of a model. Annals of Statistics. 1978; 6(2):461–464.

6. Efron B, Tibshirani R. An Introduction to the Bootstrap: Boca Raton, FL, 1993.

7. Ramsay JO. Monotone Regression Splines in Action. Statistical Science. 1988; 3(4):425–441.

8. A A. Categorical Data Analysis, 1990.

9. Cox D. Regression models and life tables (with discussion), 1972.

10. Kalbfleisch J, Prentice R. The statistical analysis of failure time data, second edition: Wiley, 2002.

11. Draper NaS, H. Applied Regression Analysis. 2d Edition, New York ed, 1981.

12. Team RC. R: A language and environment for statistical computing. 2022 [cited; Available from: <https://www.R-project.org/>
